# Characterization and Cytotoxicity of PM_<0.2_, PM_0.2–2.5_ and PM_2.5–10_ around MSWI in Shanghai, China

**DOI:** 10.3390/ijerph120505076

**Published:** 2015-05-12

**Authors:** Lingling Cao, Jianrong Zeng, Ke Liu, Liangman Bao, Yan Li

**Affiliations:** 1 Shanghai Institute of Applied Physics, Chinese Academy of Sciences, Shanghai 201800, China; E-Mails: caolingling@sinap.ac.cn (L.C.); zengjianrong@sinap.ac.cn (J.Z.); liuqingke@sinap.ac.cn (K.L.); baoliangman@sinap.ac.cn (L.B.); 2 School of Physics, University of Chinese Academy of Sciences, Beijing 100049, China

**Keywords:** coarse particles, Fine particles, ultrafine particles, metallic elements, cytotoxicity, ROS generation

## Abstract

*Background*: The potential impact of municipal solid waste incineration (MSWI), which is an anthropogenic source of aerosol emissions, is of great public health concern. This study investigated the characterization and cytotoxic effects of ambient ultrafine particles (PM_<0.2_), fine particles (PM_0.2–2.5_) and coarse particles (PM_2.5–10_) collected around a municipal solid waste incineration (MSWI) plant in the Pudong district of Shanghai. *Methods*: Mass concentrations of trace elements in particulate matter (PM) samples were determined using ICP-MS (Inductively Coupled Plasma Mass Spectrometry). The cytotoxicity of sampled atmospheric PM was evaluated by cell viability and reactive oxygen species (ROS) levels in A549 cells. *Result*: The mass percentage of PM_0.2–2.5_ accounted for 72.91% of the total mass of PM. Crustal metals (Mg, Al, and Ti) were abundant in the coarse particles, while the anthropogenic elements (V, Ni, Cu, Zn, Cd, and Pb) were dominant in the fine particles. The enrichment factors of Zn, Cd and Pb in the fine and ultrafine particles were extremely high (>100). The cytotoxicity of the size-resolved particles was in the order of coarse particles < fine particles < ultrafine particles. *Conclusions*: Fine particles dominated the MSWI ambient particles. Emissions from the MSWI could bring contamination of anthropogenic elements (Zn, Cd and Pb) into ambient environment. The PM around the MSWI plant displayed an additive toxic effect, and the ultrafine and fine particles possessed higher biological toxicity than the coarse particles.

## 1. Introduction

Atmospheric particulate matter (PM) is one of the main environmental pollutants in China. Emissions of PM frequently cause smog and haze in a large number of cities all over the whole country. Epidemiological studies have shown that long-term exposure to high concentrations of PM can cause various respiratory and cardiovascular diseases [1,2,3]. Several studies have hypothesized that atmospheric PM of smaller sizes (*i.e.*, PM_2.5_), due to their larger surface area to mass ratio, can be more potent than larger ones (*i.e.*, PM_10_) in inducing cytotoxic and/or inflammatory responses in various lung models [4,5]. Moreover, PM_2.5_ has been listed as an important air pollutant due to its potential effects of bioaccumulation, oxidation and inflammation in the human body [6,7,8]. Recent research has found that the ultrafine particles only account for a small proportion of PM, however, they contain many soluble metallic elements that can induce macrophage reactive oxygen species (ROS) activity [5]. Generally, the adverse effects of PM on human health are potentially associated with size, surface area and chemical composition, such as trace elements and black carbon [9]. Therefore, the relationships between specific PM and health conditions still remains as an active and important topic of aerosol research.

Shanghai, a commercial mega-city with a population of over 20 million, is facing complicated atmospheric environmental problems. There are various pollution sources [10,11,12,13] such as vehicle exhaust, industrial activities and municipal solid waste incineration (MSWI). For reasons of reducing the volume waste and allowing for recovery of energy, incineration is playing a favorable role in wastes management. A key indicator in the Chinese National Five-Year Plan stipulates that the national rate of incinerated municipal solid waste should reach 35% by the year 2015. Since the first MSWI was established in Guangdong Province in 1988, it is estimated that the number of incinerators in China will increase to 321 by the year 2015. In Shanghai, there are three large-scale MSWIs in operation. Although the best available techniques of flue gas cleaning system are used in MSWI, the hazardous air pollutants (HAPs) cannot be removed completely and a considerable part of them are released into the ambient atmospheric environment. The national total emissions of HAPs from MSWI have increased rapidly from 25.97 to 179.26 kilo-tons during the period 2003–2010 with the development of the incineration industry in China [14]. Heavy metals associated with PM are a major part of HAPs emitted from MSWI exhaust. Some studies conclude that MSWI contributes directly to heavy metal contamination of the surrounding air. Hu *et al.* found that metallic elements, particularly V, Cr, Mn, Ni and Cd, in the local airborne particles are highly influenced by the stack emission of MSWI in Taiwan [15]. Airborne Zn, Cd and Sb are attributed the refuse combustion in many areas of Washington, D.C. [16]. In Japan, the major metallic elements of Pb, Cd, Zn and As in PM were highly associated with MSWI emissions [17]. Although it is easy to foresee that with the increase in the number of incinerators, abundant toxic trace elements will inevitably be emitted into the surrounding atmosphere. Only a few studies in China have focused on the characterization and toxicity of ambient PM around the MSWI, which directly pose health risks to the surrounding residents.

The purpose of this study was to determine the metallic elements of ambient PM samples surrounding MSWI and investigate their cytotoxicity. Size-segregated particles were collected around a MSWI in Pudong district, Shanghai. The mass size distributions of metallic elements in the PM (ultrafine, fine and coarse particles) were measured to discuss the characteristics of PM. A549 cells were exposed to sampled particles of different sizes, and the cell viability and ROS levels in treated A549 cells were detected to determine their potential *in vitro* cytotoxicity.

## 2. Materials and Methods

### 2.1. Sampling Site and Samples

**Figure 1:**
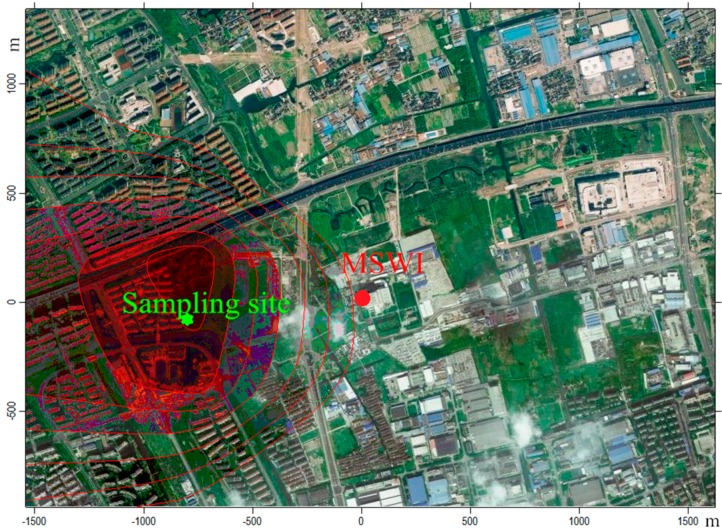
The concentration distribution of particulate matter (PM) from the municipal solid waste incineration (MSWI) calculated by AERMOD (atmospheric dispersion modeling) (prevailing wind direction: east, average wind speed: 3.4 m/s and temperature: 21 °C) and the location of the sampling site.

The incinerator located in the Pudong district of Shanghai is the first Chinese kiloton waste incineration power plant. It possesses a steady treatment capacity of about 1000 t/d with typical type of incineration technology and gas purification system. There are limited large industrial activities in Pudong, which is located in the eastern coastal area of Shanghai and far from the industrial areas. The AERMOD, a steady-state plume model, was used to calculate the PM dispersion of the MSWI as a point source. The model is widely used for the assessment of pollution concentrations from different types of stationary industrial sources [18]. The required inputs for simulation include emission parameters and meteorological data. The emission parameters of this MSWI plant were as follows: height (80 m), diameter (1.6 m), exit velocity (14.5 m/s), exit temperature (150 °C) and PM emission rate (0.28 t/d). The ground-level data (prevailing wind direction is east, average wind speed is 3.4 m/s and temperature is 21 °C) in May/June were obtained from the Weather Underground website (www.wunderground.com) and the upper-air data were obtained from NOAA/ESRL. The daily PM dispersion from this MSWI plant was simulated, and is shown in Figure 1.

Simulation results revealed that the PM emissions from MSWI had formed a concentration gradient in the modeling area. The deeper color in Figure 1 indicates a higher concentration that appeared in the range of 800–1000 m downwind from the MSWI, with the inner region being the highest polluted area. In order to collect as much PM from the MSWI as possible, the PM sampling site (840, −124) was set at 850 m downwind the MSWI site (0, 0), which was located in a residential building surrounded by many flat farmlands and residential areas with no other large apparent emission sources nearby (Figure 1). A low-pressure multistage impactor (DLPI, Dekati Ltd., Kangasala, Finland) was used to collect size-segregated PM samples. The 50% cut-off aerodynamic diameters (*D_a_*) of DLPI (13 stages) are 0.0283, 0.0545, 0.0911, 0.121, 0.261, 0.380, 0.611, 0.945, 1.59, 2.38, 3.98, 6.47, and 9.92 μm, working at a flow rate of 30 L/min. The fractions of particles collected on the impactor stages 1–4 (*D_a_* < 0.2 μm) are referred to as the ultrafine particles, while the portions on the impactor stages 5–9 (0.17 μm < *D_a_* < 2.5 μm), and on the impactor stages 10–13 (*D_a_* > 2.5 μm) are respectively referred to as the fine and the coarse particles. The particles were collected on the polytetrafluoroethylene (PTFE) filters (ADVANTES, 25 mm diameter, 0.2 μm pore size). The sampling campaign was carried out from May 15 to June 16, 2012, according to the meteorological conditions used in the simulation. Usually, the concentration of PM was relative low during the period of May-June in Shanghai [19], which favored the collection of PM from MSWI. The actual local ambient temperature, prevailing wind direction and wind speed during the sampling periods were 21–27 °C, east and 3.0–5.4 m/s, respectively. Four sets of PM sample were collected with each sample being collected for 72 h. Three sets of the sampled PM were used for chemical analysis and one set was used for *in vitro* experiments.

### 2.2. Chemical Analysis

The PTFE filters were stored in a drying cabinet and balanced in relative humidity of 40%–45% at room temperature for 24 h for weighing both before and after sampling. Then PTFE filters were weighed using an electronic balance (resolution ±1 μg, Crystal 250, Gibertini, Italy).

*Elemental analysis:* The filter samples were digested using a mixed digestion solution (6 mL 67% HNO_3_ + 2 mL 30% H_2_O_2_ + 0.5 mL 40% HF for each sample) by heating in a microwave oven (Excel, PreeKem, China). Microwave digestion consisted of three steps: (1) heating to 220 °C in 19 min gradually by increasing the power to 1500 W; (2) remaining at 220 °C for 30 min at 1500 W; (3) cooling down for 1 h. After digestion, the solutions were heated to evaporate the acid, and then transferred and diluted with deionized water (18.2 MΩ, MiliQA10, Milipore, Billerica, MA, USA) to a final volume of 10 mL. The mass concentrations of Na, Mg, Al, K, Ti, V, Cr, Mn, Fe, Ni, Cu, Zn, Cd, and Pb in the PM samples were quantified by ICP–MS (NexION 300 D, PerkinElmer, Waltham, MA, USA). The standard addition method was used for the determination of these elemental concentrations by preparing a series of mixed calibration solutions (AccuStandard, New Haven, CT, USA) at different concentrations (1, 5, 10, 20 ng/mL). A quantity of 10 ng/mL ^115^In was added online as the internal standard used to compensate for the interference from non-spectroscopic effects. The determination of Na, Mg, Al, K, and Fe concentrations was performed using the kinetic energy discrimination effect (KED) mode. The concentrations of the other metals were obtained using the standard mode. The accuracy of the analytical procedure was confirmed using a standard reference material SRM 1648 (Urban Particulate Matter, National Institute of Standards and Technology, Gaithersburg, MD, USA).

#### Cell Culture and Exposure

*Cell culture:* The A549 cell line, a popular model extensively referenced in the literature on toxicology [20,21], is a human lung adenocarcinoma derived by an explant culture from the peripheral airways of a Caucasian male with lung cancer. The A549 cells were purchased from the China Center for Type Culture Collection (Wuhan, China). The cells were maintained in a dulbecco’s modified eagle medium (DMEM) (Thermo Fisher Scientific, Shanghai, China) supplemented with 10% FBS, 100 μg/mL penicillin and 100 μg/mL streptomycin in an incubator aerated with 5% CO_2_ at 37 °C and passed at 80% of confluence. The PM samples were dispersed in a serum-free DMEM, and sonicated for 3 × 10 s before application. The suspension was co-incubated with A549 cells for 6 h. Those A549 cells cultured without PM served as the control group.

*Cell viability assay:* Absorbance was quantified at 450 nm using a microplate reader (Synergy2, Bio-tek, Winooski, VT, USA). Cell viability was determined by cell counting kit-8 (CCK-8, Beyotime Institute of Biotechnology, Jiangsu, China) according to the manufacturer’s instructions, which were based on the conversion of water-soluble tetrazolium salt, WST-8 [2-(2-methoxy-4-nitrophenyl)-3-(4-nitrophenyl)-5-(2,4-disulfophenyl)-2H-tetrazolium, monosodium salt] to a water-soluble formazan dye upon reduction by dehydrogenases in the presence of an electron carrier [22]. A549 cells were seeded in 96-well culture plates with 5 × 10^3^ cells in 100 μL DMEM per well. After 26 h of cell attachment, the cells were treated with PM for 6 h. Then, 10 μL WST-8 solution was added to each well, and the cells were further incubated at 37 °C for 1 h in the incubator. Cells without any treatment served as the control. Absorbance was quantified at 450 nm using a microplate reader (Synergy2, Bio-tek, Winooski, VT, USA). The experiment was repeated three times. Cell viability was expressed as a percentage of the control culture’s value: (Test group A/control A) × 100%.

*Measurement of intracellular ROS formation:* Intracellular ROS lever was measured by the fluorescent dye, 2,7-dichlorofluorescein diacetate (DCFH-DA), which is taken up by the cells, and on deacetylation forms a non-fluorescent DCFH, which is converted into fluorescent DCF when oxidized by cellular ROS. A549 cells incubated with particle samples (ultrafine, fine and coarse particles) were washed three times with 0.1M PBS and fixed with 4% formaldehyde for 15 min. Then, the cells were washed with PBS again. After that, a permeabilizing buffer (10.3 g of sucrose, 0.292 g of NaCl, 0.06 g of MgCl_2_, 0.476 g of Hepes buffer, 0.5 mL of Triton-X-100, in 100 mL of water, pH 7.2) was added for 5 min, and then incubated with 1% bovine serum albumin/PBs at 37 °C for 5 min. Afterward, the cells were stained with 25 μmol·L^−1^ DCFH-DA for 10 min. The resultant fluorescence intensity was measured by a laser scanning confocal microscope (TCS SP2, Leica Ltd., Co, Wetzlar, Germany) at 485 nm excitation wavelength and 530 nm emission wavelength [23,24].

### 2.3. Statistical Analysis

The data for the mass concentration of the PM and the metallic elements in it were analyzed by Excel. The cell viability assay data was analyzed by a one-way ANOVA followed by Dunnett’s *t*-test using SPSS software for comparisons between groups. *p* < 0.05 was considered significant when compared to the control. Moreover, all numerical data were presented as the mean ± standard deviations (S.D.).

## 3. Results and Discussion

### 3.1. Characterization of Particulate Matters

**Figure 2:**
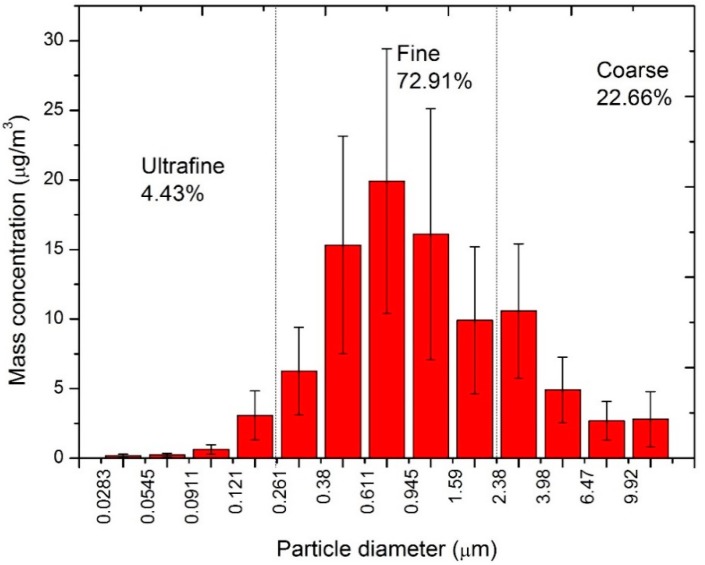
Average mass concentrations (μg/m^3^) of the 13-size particles of PM samples around MSWI.

The average mass concentrations of PM are shown in Figure 2. From 15 May to 16 June 2012, the average PM_10_ sampled in Pudong was 92.58 ± 47.70 μg/m^3^, in which ultrafine, fine and coarse particles were 4.10 ± 2.38, 67.51 ± 34.77 and 20.98 ± 10.55 μg/m^3^, respectively. Most of the particles were fine. Furthermore, the PM_10_ concentration in the samples exceeded the daily ambient air quality PM_10_ standard I (50 μg/m^3^, GB3095-2012) in China, while the concentration of PM_2.5_ was 71.61 ± 37.15 μg/m^3^, which was about two times higher than the PM_2.5_ standard I (35 μg/m^3^, GB3095-2012). However, the PM_2.5_ concentration was in the range of the reported values in most districts of Shanghai, such as 84.1 μg/m^3^ in Zabei [19], 82.5 μg/m^3^ in Jiading [19], 103.07 μg/m^3^ in Baoshan [25], and 62.25 μg/m^3^ in Putuo [25]. These results suggested that the MSWI emissions had not significantly contributed to the collected PM_2.5_ samples. The size-resolved particulate mass distribution derived from the 13-stage impactor showed a maximum in the range of 0.611~0.945 μm in fine particles. The ultrafine particles accounted for 4.43% of the total PM_10_ mass, while the fine particles accounted for 72.91%, and the coarse particles accounted for 22.66%, indicating that the fine particles had dominated the ambient particles of the incinerator in Shanghai.

The metallic elemental concentrations of the PM samples determined by ICP-MS are shown in Table 1. The concentrations of Mg, Al and Ti in coarse particles was higher than those in the fine and ultrafine ones, as a result of these elements from natural origin sources [10,19]. For Na and Fe, their concentrations in the fine and coarse particles were similar, much higher than those in ultrafine particles. Interestingly, the concentration of K in the fine particles was 4.6 and 8.8 times of that in the coarse and ultrafine particles, respectively. Since K in the fine particles was primarily related to biomass burning activities [26], and is frequently present in the kitchen waste, which is a major component of incinerated garbage in China, it can be deduced that K was highly associated with the MSWI emissions. The concentrations of V, Mn, Ni, Cu, Zn, Cd and Pb in the fine particles were much higher than those in the coarse and ultrafine particles, and the concentration of Cr in the fine particles was slightly higher than that in the coarse particles. Generally, these heavy metals were primarily associated with anthropogenic sources, such as industrial emissions, traffic sources, coal combustion, and waste burning [15,19,27,28]. As a result, these elements tended to concentrate in fine particles.

**Table 1:** Average (± standard error) mass concentrations (ng/m^3^) and EFs of metallic elements in ultrafine, fine, coarse particles.

Element	Ultrafine Particles		Fine Particles		Coarse Particles	
	Mass Concentrations	EFs	Mass Concentrations	EFs	Mass Concentrations	EFs
**Na**	37.72 ± 13.31	5.8	304.25 ± 118.55	8.2	244.85 ± 125.48	4.4
**Mg**	13.57 ± 12.53	2.7	84.08 ± 17.99	2.9	108.83 ± 48.15	2.5
**Al**	39.65 ± 9.87	1.0	226.43 ± 108.59	1.0	355.61 ± 302.19	1.0
**K**	64.79 ± 40.44	6.5	575.99 ± 393.89	10.2	124.72 ± 83.65	1.4
**Ti**	8.57 ± 2.95	3.5	25.65 ± 10.24	1.8	36.39 ± 21.12	1.7
**V**	1.98 ± 0.44	39.1	11.41 ± 3.79	39.4	1.03 ± 0.75	2.3
**Cr**	6.88 ± 1.24	173.6	13.97 ± 2.86	61.7	11.96 ± 5.88	31.7
**Mn**	1.82 ± 0.31	5.9	21.41 ± 9.15	12.1	8.15 ± 6.20	3.0
**Fe**	81.43 ± 26.40	4.6	343.50 ± 148.34	3.4	319.15 ± 249.92	2.0
**Ni**	2.61 ± 0.64	154.9	13.87 ± 8.78	143.8	6.78 ± 5.61	45.4
**Cu**	1.45 ± 0.42	94.5	8.02 ± 3.68	91.4	2.32 ± 1.34	17.5
**Zn**	10.29 ± 2.90	224.1	89.16 ± 30.11	340.0	10.87 ± 4.43	25.3
**Cd**	0.03 ± 0.04	390.9	0.35 ± 0.21	790.8	0.01 ± 0.01	10.5
**Pb**	3.19 ± 1.43	225.8	35.25 ± 21.85	437.1	3.02 ± 2.38	32.6

In order to assess the extent of the contribution of anthropogenic emissions to atmospheric elemental levels, the enrichment factors (*EFs*) for each metal in the ultrafine/fine/coarse particulates were analyzed. Each *EF* listed in Table 1 was calculated using the following equation:

(1) $$EF_{i}\text{=}\frac{{\left(M_{i}\text{/}M_{r}\right)}_{\text{aerosol}}}{{\left(M_{i}\text{/}M_{r}\right)}_{\text{crust}}}$$

where *M_i_* is the concentration of the element in aerosol or crust, and *M_r_* is the abundance of Al in the aerosol or crust [28]. Generally, if *EFs*< 10, chemical elements are emitted from natural or anthropogenic sources [28]. According to the results, the *EFs* of the crustal elements (Na, Mg Al, Ti, and Fe) in all three size ranges of the particles were less than 10, indicating that these elements were mostly from natural sources. However, the *EFs* of K and Mn were a little higher than 10 in the fine particles, but lower than 10 both in the coarse and ultrafine particles. This meant that K and Mn in the fine particles had multiple sources. V, Zn, Cd and Pb had significantly higher *EFs* in the ultrafine and fine particles than in the coarse particles, suggesting that these elements had been attracted to the ultrafine and fine particles. The *EFs* of Zn, Cd and Pb in the fine and ultrafine particles were much higher than 100, revealing that such heavy metal pollution was significant in the fine and ultrafine particles. These semi-volatile elements are released into the atmosphere in the effluents of most combustion processes, such as non-ferrous metal melting, coal combustion and traffic engine burning [10,11,12,25]. Our previous study had found that these elements were enriched with extremely high *EFs* (94.7, 1947.6 and 263.8 for Zn, Cd and Pb, respectively) in the fly ash of this MSWI [29]. Considering that the sampling site of this work was located downwind of the MSWI and there were no other large apparent emission sources nearby, it can be deduced that these elements (Zn, Cd and Pb) were highly associated with the emissions of MSWI. As a matter of fact, Zn, Cd and Pb can be easily found in MSWI emissions. In Japan, 60% of Pb, 94% of Cd and 78% of Zn in urban PM samples collected near MSWI sites were attributed to MSWI emissions [15]. Cd was also frequently found in the stack emissions from MSWI in Taiwan [17]. In Shanghai, Pb in the particles of 0.4–1.6 μm, associated with Cl, was found to have mostly been emitted from waste incineration [13].

**Figure 3:**
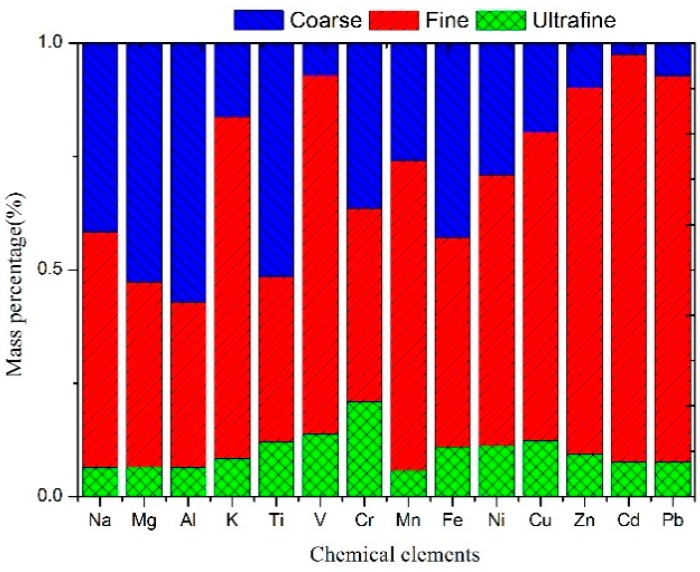
The mass ratios of the metallic elements in the coarse particles (blue), fine particles (red) and ultrafine particles (green).

The mass ratios of elements of different particle sizes are illustrated in Figure 3. More than 50% of Na, Mg, Al, Ti and Fe were found in the coarse particles, suggesting that crustal elements had been distributed mainly in the coarse particles. More than 50% of K, Mn, Ni, Cu, Zn, Cd and Pb were found in the fine particles, indicating that the anthropogenic elements were more predominant in the fine particles. Relatively, few metallic elements were observed in the ultrafine particles; however, ultrafine particles, which are more easily inhaled and seriously impact human health, have been receiving increased attention [30,31].

### 3.2. Cytotoxicity of Particulate Matters

**Figure 4:**
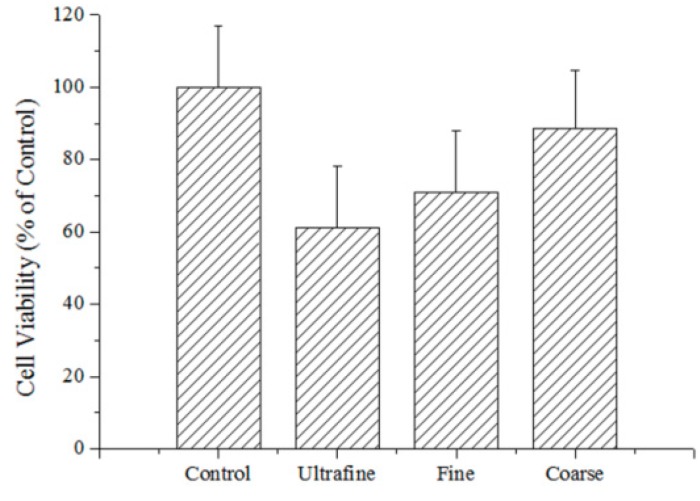
Viability of PM with different sizes to A549 cells after 6 h exposure (*p* < 0.05).

In order to evaluate the toxic effects of atmospheric PM, cell viability was detected by CCK-8 assay. A549 cells were treated with ultrafine, fine and coarse particles for 6 h. The detection results of cell viability are shown in Figure 4. The cell viability had slightly decreased after the treatment of the coarse particles, while it had significantly decreased after the treatment of the fine and ultrafine particles when compared to the control groups.

**Figure 5:**
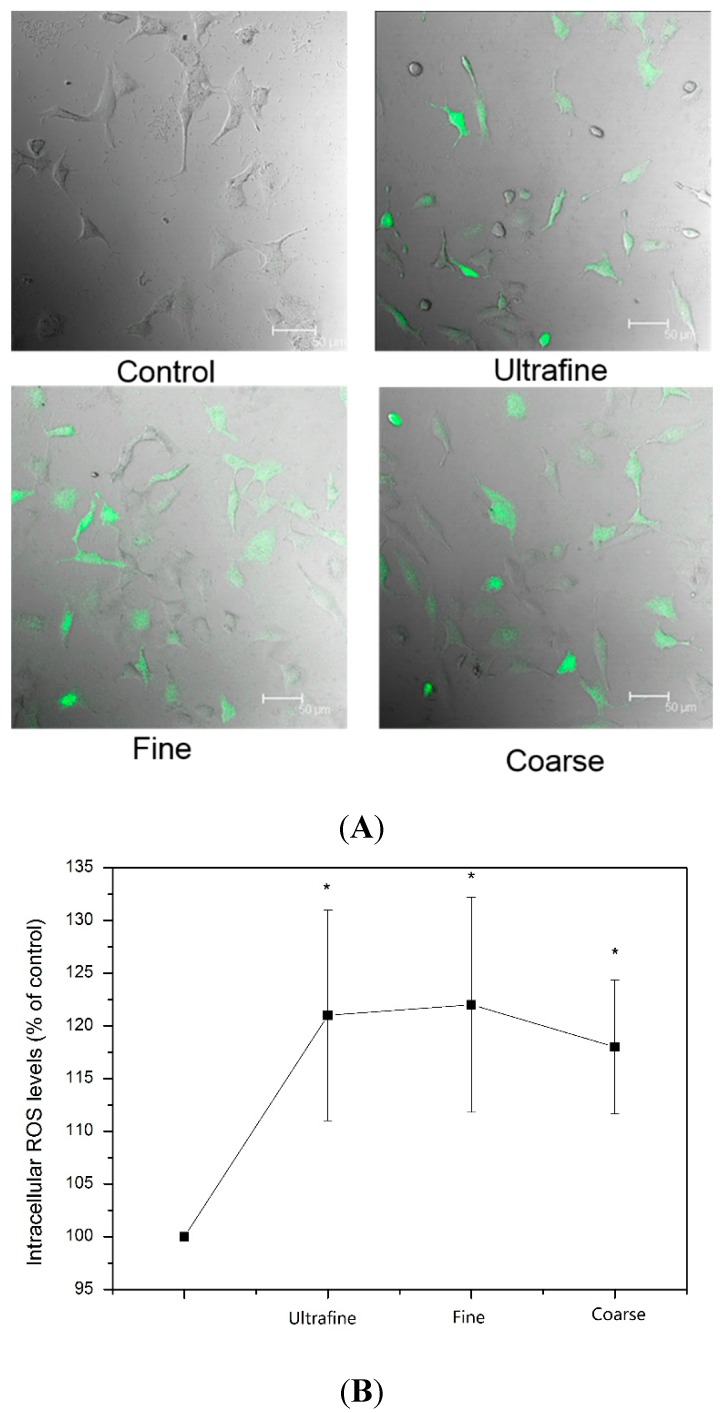
(**A**) LSCM (Laser Scanning Confocal Microscope) images and (**B**) intracellular reactive oxygen species (ROS) levels of A549 cells with PM samples (ultrafine, fine and coarse particles).

To get a closer insight into the possible toxic mechanism of the PM samples, the intracellular ROS levels were determined by using the DCFH-DA probe. As shown in Figure 5, the ROS levels were elevated in A549 cells cultured with PM samples (coarse, fine and ultrafine particles). Very weak fluorescence was detected in the cells without treatment, while the obvious green fluorescence was observed after the treatment of the PM samples. Figure 5A shows that the generation of ROS in all samples with PM had significant differences when compared to the control group. The ROS levels of the A549 cells were promoted by about 20% after culturing with the PM samples. Normal A549 cells were present as long fusiforms, which could easily be seen in the LSCM images. Such types of cells could also be found in the group cultured with the coarse particles, but hardly seen in the group cultured with the fine and ultrafine particles. Furthermore, the circular cells were easily found in the group cultured with ultrafine particles, which might be the apoptosis of the cells. It can be concluded that the existence of PM had increased the ROS levels and reduced cell viability, which was consistent with the results of the tests for cell viability.

Particle size, composition and specific emission sources of atmospheric PM samples have different biological effects [32,33]. In this study, the size distribution, metallic composition and *in vitro* toxicology were determined.

The coarse particles had significantly induced the generation of intracellular ROS levels, but not the apoptosis of the cells, which might due to the fact that these sizes of particles could not penetrate into the cells. However, these particles could induce the free radicals, which only caused the ROS generation with the cell self-defense capability. The coarse particles, with diameters >2.5 μm, had hardly penetrated into the cells directly and induced a decrease in cell viability. Meanwhile, the coarse particles, containing 50% of natural elements, such as Na, Mg, Al, Ti and Fe, might have induced the free radicals. This would have resulted in intracellular ROS generation with cell self-defense capability.

The main proportion of the PM samples was the fine particles with diameters of 0.261 μm to 2.38 μm. Such particles can enter the A549 cells through endocytosis [34]. In our PM samples, the larger proportion of the elements from anthropogenic sources, V, Ni, Cu, Zn, Cd and Pb were present in the fine particles. After these particles [32] had entered the cell into the cytoplasm, lysosomes and autophagosomes, the toxic elements Cu, Cd, Zn and Pb were able to induce the generation of ROS and decrease cell viability.

The ultrafine particles were only about 4.4% of the total mass concentration, but it had induced a significant decrease in cell viability. Furthermore, it had also induced the generation of ROS intracellular levels and changes in cell morphology. This result was due to the ultrafine particles having a much higher number concentration and a larger surface area [32] than did the larger particles. Nanoparticles with diameters <100 nm could enter into cells by endocytosis or cross the cell membrane directly [35,36]. The ultrafine particles, with diameters <100 nm, could penetrate the cytoplasm, lysosomes and autophagosomes more easily thus bring high toxicity to A549 cells.

## 4. Conclusions

In this study, the total mass concentrations of PM samples collected in the vicinity of a MSWI plant in Pudong district, Shanghai were 92.58 ± 47.70 μg/m^3^, in which ultrafine, fine and coarse particles were 4.10 ± 2.38, 67.51 ± 34.77, 20.98 ± 10.55 μg/m^3^, respectively. The fine mass concentration ratio was as high as 72.91%, indicating that the MSWI ambient PM was dominated by the fine particles. Crustal metal elements (Mg, Al, and Ti) were predominant in the coarse particles, while the anthropogenic metal elements (V, Ni, Cu, Zn, Cd and Pb) were predominately concentrated in the fine particles. Zn, Cd and Pb in the fine and ultrafine particles were heavily polluted with EFs much higher than 100. These elements were highly associated with the emissions of MSWI. The toxic responses of the A549 cells to the sampled particles of different sizes were markedly different. The toxicity level of PM on cells both depended on the particle size and the content of toxic composition in particles, especially the concentration of toxic metal elements. The PM samples around the MSWI had induced an increase in intracellular ROS levels with decreasing particle sizes in the A549 cells, and the cell viability of the size-resolved particles followed the order of coarse particles > fine particles > ultrafine particles, indicating that cytotoxicity of the ultrafine and fine particles were much more severe than the coarse particles. The toxicity level of PM on cells both depended on the particle size and the content of toxic composition in particles, especially the concentration of toxic metal elements.
